# Emotional Intelligence and Psychological Well-Being in Adolescents

**DOI:** 10.3390/ijerph16101720

**Published:** 2019-05-16

**Authors:** Joan Guerra-Bustamante, Benito León-del-Barco, Rocío Yuste-Tosina, Víctor M. López-Ramos, Santiago Mendo-Lázaro

**Affiliations:** 1 Department of Psychology, Faculty of Teacher Training College, University of Extremadura, 10071 Cáceres, Spain; joangb@unex.es (J.G.-B.); bleon@unex.es (B.L.-d.-B.); vmlopez@unex.es (V.M.L.-R.); 2 Department of Educational Science, Faculty of Teacher Training College, University of Extremadura, 10071 Cáceres, Spain; rocioyuste@unex.es

**Keywords:** adolescent, well-being, emotional intelligence, happiness

## Abstract

The present study aimed to analyze the association between of the dimensions of emotional intelligence (attention, clarity, and repair) and different levels of perceived happiness (low, medium, and high) in adolescents. The sample consists of 646 students in the first, second, third, and fourth years of Secondary Education, 47.5% females and 52.5% males, between 12 and 17 years of age. The instruments used were the Spanish version of the Trait Meta Mood Scale-24 Questionnaire to measure perceived emotional intelligence and the Oxford Happiness Questionnaire. Multinomial logistic regression analysis and receiver operating characteristic (ROC) curve analysis were performed. The results suggest that as the capacity of understanding and regulation of emotional intelligence increases, happiness also increases. Adolescence is seen as an ideal time in life to encourage the development of emotional capacities that contribute to the greater happiness of individuals. In this way, the present study stresses the need to carry out practices leading to improvements in the adolescents’ emotional intelligence and therefore increase their happiness and emotional well-being.

## 1. Introduction

The study of happiness and emotional well-being in young people has expanded exponentially in recent years. Psychology has traditionally focused on unhappiness and paid little attention to positive aspects of human potential [1]. This approach has been evident when studying adolescence, since this period of life implies many changes and it has been long described as a moment of stress and difficulties [2]. This conception of adolescence is currently fairly different for studies do not only describe the adolescent as a source of problems but also as a valuable asset in a development process [3,4]. This change took place with the arrival of positive psychology, as one of its objectives is to promote psychological research and practice in such areas as positive traits (strengths), positive emotions, and their contribution to well-being [5].

### 1.1. Happiness or Psychological Well-Being

As for the study of happiness, it is essential to point out that there is no consensus about how to define it. One of the most accepted theoretical approaches states that the construct happiness refers to an emotional and cognitive type of psychological state [6], a positive affective component in which positive emotions and the subjective interpretation of well-being are fundamental [6,7,8,9,10,11,12].

On a theoretical level, the debate on happiness has two main approaches: 1) the hedonic approach, that affirms that happiness is the presence of positive affection and the absence of negative affection; and 2) the eudaimonic approach, that states that happiness is the consequence of full psychological functioning by means of which the person develops his or her potential [13]. In line with eudaimonism, it is noteworthy to mention the psychological well-being multidimensional model [14], focused in the fulfillment of human potential through six key features: autonomy, environmental control, personal growth, positive relationships with others, purpose in life, and self-acceptance [15]. Both approaches can be integrated in the “three dimensions of happiness” model [1] which are: 1) a pleasant life, understood as a pleasant feeling towards past, present and future; 2) a committed life, by using positive individual features, including character strengths and talents; and 3) a meaningful life, which means to serve and to belong to positive institutions. Subsequently, this model favored the appearance of 24 Strengths Model [16] which focuses on studying happiness in strengths and virtues.

Accordingly, they reinforce the idea of the existence of factors that determine happiness [17]. Then we find the Science of Happiness [12] which claims that happiness can be increased by the individual himself by means of certain activities. For that matter, such a vital period as adolescence is the ideal moment to increase it. In recent years, different theoretical approaches have defended a positive comprehension of adolescence, a crucial stage characterized by plasticity, the acquisition of competences and the achievement of satisfactory levels of well-being and positive adjustments [17]. It is a time when the capacity to appreciate satisfaction with life and well-being increases in a critical and conscious way [18]. Specifically, teaching adolescents to be happy functions with three main goals: as an antidote against depression, as a means of increasing life satisfaction, and as a way to enhance learning and creative thought [19].

### 1.2. Emotional Intelligence

One of the variables that could help to this increase of happiness during adolescence can be emotional intelligence [20]. There are two relevant models of emotional intelligence: Mixed Models and Ability Model. Mixed Models state that emotional intelligence is a compendium of stable personality features, socio-emotional competences, motivational aspects, and different cognitive abilities [21,22,23]. On the other side we find the Ability Model [24] which considers emotional intelligence as an ability focused on emotional information processing [25]. Ever since Model of Emotional Intelligence, this construct is defined as a type of social intelligence that involves the ability to monitor one’s own and others’ emotions, to discriminate among them, and to use the information to guide one’s thinking and actions [24]. Subsequently, said authors included in their definition abilities related to cognitive and emotional clarity, perception, and repair that could generate feelings that eased thinking and abilities of cognitive and emotional regulation [26]. In order to measure this construct, they designed questionnaire TMMS-24, which assesses Perceived Emotional Intelligence through three factors: attention to emotions (capability to feel and express feelings properly), emotional clarity (capability to understand the own emotional states), and emotional repair (capability to correctly regulate emotional states).

### 1.3. Happiness or Psychological Well-Being and Emotional Intelligence

Scientific literature highlights the major role of emotional intelligence when determining individual happiness [20]. Numerous researchers have related emotional intelligence with psychological constructs that are closely associated with happiness, such as subjective well-being [27,28], higher rates of positive emotional states and decrease of negative emotional states [29], satisfaction with life [20,30,31,32], better psychological functioning and social competence [33], and better social relations; and negative associations with loneliness [34,35,36,37,38,39,40]. Other studies have focused on the relationship between emotional intelligence and variables connected with well-being in young people, such as physical and mental health [41,42,43] and perception of stress [44]. There is therefore clear evidence that capacities of emotional intelligence predict aspects related to personal well-being and a positive relation between life satisfaction and subjective happiness [45,46].

For this matter, Hills and Argyle [47] composed the Oxford Happiness Questionnaire, which evaluates subjective happiness from these psychological dimensions, including items focused on life satisfaction, positive emotions, physical and mental health, or social relationships.

More specifically, studies made from mixed models note that the trait emotional intelligence is a constellation of capacities and self-perceived attitudes related with emotion [48]. In this regard, different studies note the existence of a positive correlation between emotional intelligence as a trait and perceived happiness [49,50]. On the other hand, from the ability model, research based on Spanish adolescent subjects shows that the abilities of clarity and repair are positively correlated with life satisfaction whereas attention correlates negatively in adolescents [51]. In the same way, the dimensions of emotional recognition and expression, and the control of emotions mediate in the relationship between fully dispositional mindfulness and subjective happiness [52]. However, it should be considered that self-perceptions and attitudes associated with people’s emotions—such as emotional regulation, relationship skills, and social competence—determine variation in happiness to a large degree [50]. Henceforth, research shows that emotional intelligence abilities imply a skill that allows adolescents to guide their thoughts and ponder over their emotions, helping them to improve their well-being levels [53]. These studies suggest that important interventions may be performed to promote flourishing and happiness, enhancing emotional intelligence through specific training [54].

The present study seeks to analyze in a sample of adolescents, the association between of the dimensions of emotional intelligence (attention, clarity, and repair) and different levels of perceived happiness (low, medium, and high). It will also identify the sensitivity and the ability to distinguish scores obtained in the Spanish version of the questionnaire Trait Meta Mood Scale [55], from which high happiness is more likely to exist. 

## 2. Materials and Methods

### 2.1. Participants

The sample consists of 646 students in the first, second, third, and fourth years of Secondary Education, 47.5% females and 52.5% males, between 12 and 17 years of age. The sampling was carried out by selecting eight schools in the Community of Extremadura (Spain) at random.

### 2.2. Instruments

#### 2.2.1. Trait Meta Mood Scale

The Spanish version of the questionnaire Trait Meta Mood Scale (TMMS-24) [55] has been used to evaluate perceived emotional intelligence. The questionnaire is formed by 24 items with a Likert-type five-point answer scale (1 = Do not agree, 5 = Totally agree). Three dimensions are evaluated (eight items per dimension): attention (ability to feel and express feelings appropriately); clarity (understanding of emotional states); and repair (appropriate emotional regulation). Each dimension can be classified into three traits depending on the score: Attention; 1) Attention should be improved; 2) Adequate attention; 3) Excessive attention: Clarity; 1) Clarity should be improved; 2) Adequate clarity; 3) Excellent clarity: Repair; 1) Repair should be improved; 2) Adequate repair; 3) Excellent repair. The internal consistency measured with Cronbach’s alpha was 0.826 for attention, 0.825 for clarity, and 0.833 for repair.

#### 2.2.2. Oxford Happiness Questionnaire

The Oxford Happiness Questionnaire (OHQ) [47]. The objective of this questionnaire is to measure happiness in general, i.e., psychological well-being. A series of statements about happiness are given and the participants indicate their degree of agreement with each one. In psychometric terms, it consists of 29 items or 29 potential sources of happiness and the participants consider the extent to which they form part of their experiences. It employs a six-point Likert-type scale (1 = I totally disagree, 6 = I totally agree). The lowest score that can be obtained is 1 (if Answer 1, ‘I totally disagree’ is chosen in all the statements) and the highest is 6 (if Answer 6; ‘I totally agree’ is chosen for all the statements). In this study, the internal consistency measured with Cronbach’s alpha was 0.800.

### 2.3. Procedure

The procedure followed for data collection was the administration of the questionnaires by classroom group. In the first place, the educational centers were contacted to explain the objectives of the study and request authorization for the completion of the questionnaires. We followed the ethical guidelines of the American Psychological Association regarding the informed consent of the parents, due to participants’ being underage. Likewise, anonymity in the answers, the confidentiality of the obtained data, and its exclusive use for research purposes was assured. The administration of the questionnaires was carried out during school hours; it took around 50 min. in an adequate climate and without distractions. This study was approved by the Bioethics and Biosafety Committee of the University of Extremadura (no. 0063/2018).

### 2.4. Statistical Analysis

Firstly, we submitted the data to the assumptions of independence, normality, homoscedasticity and linearity required by the classical linear model. We did not find normality or homoscedasticity in our data, so we decided to perform a multinomial logistic regression analysis. Although it may seem that transforming a variable initially classified as continuous to categorical would mean losing information, during the analysis we gain efficiency and, mostly, clarity for interpretation. Multinomial logistic regression analysis was performed to determine the degree of association between the variables being studied. The odds ratio and their 95% confidence intervals, and the receiver operating characteristic (ROC) curve were calculated. The analysis based on the ROC curves is a statistical method to determine the diagnostic preciseness of tests that use continuous scales, and are used for three specific purposes: to establish the cut-off point at which the highest sensitivity and specificity is reached; evaluate the discriminative capacity of the diagnostic test, i.e., its capacity to differentiate healthy and sick individuals; and to compare the discriminative capacity of two or more diagnostic tests that express their results as continuous scales.

## 3. Results

In order to verify that emotional intelligence is associated with happiness, multinomial logistic regression analysis included happiness as a predictor variable, grouped according to a criterion of percentiles in low, medium, and high happiness and the emotional intelligence dimensions attention, clarity, and repair as predictor variables, grouped in three categories (Table 1). Gender and age of participants were included as control variables.

Both multinomial regression analyses demonstrated a satisfactory fit, χ^2^ (16, *N* = 629) = 104.922, *p* < 0.001 (two-tailed), *ϕ* = 0.048; R Nagelkerke = 0.181, enabling correct classification in 62% of the cases.

The detailed analysis of the findings according to the different emotional intelligence dimensions shows the association between happiness and perceived intra-personal emotional intelligence, so that as clarity and repair increase, the individuals see themselves as happier, and as they decrease the individuals are less happy.

To be precise, for the result of the model with the reference category happiness (Table 2), the calculations of the parameters reveal that adequate clarity (Wald = 4.205, *p* = 0.040), adequate repair (Wald = 8.609, *p* = 0.003), adequate repair (Wald = 14.759, *p* < 0.001), and excellent repair (Wald = 8.503, *p* =0.004) are associated significantly and directly with medium happiness. In addition, adequate (Wald = 10.376, *p* = 0.001) and excellent clarity (Wald = 8.610, *p* = 0.003), and adequate (Wald = 15.997, *p* < 0.001) and excellent repair (Wald = 25.323, *p* < 0.001) are correlated directly and significantly with high happiness.

The OR calculations of the model with the reference category low happiness (Table 2) show that the probability of medium happiness is twice as high among individuals with adequate clarity, 3.4 times higher with excellent repair and 2.5 times higher with adequate repair. Similarly, the probability of high happiness is 2.7 times higher with adequate clarity, 4.1 times higher with adequate repair, 5.6 times higher with excellent clarity, and 12 times higher with excellent repair.

In addition, calculations of the parameters for the reference category high happiness (Table 2) reveal that the need to improve clarity (Wald = 8.610, *p* = 0.003), repair (Wald = 25.323, *p* < 0.001), and adequate repair (Wald = 6.281, *p* = 0.012) are associated significantly and directly with low happiness. Equally, the need to improve clarity (Wald = 9.771, *p* = 0.002) and repair (Wald = 11.861, *p* = 0.001), and adequate clarity (Wald = 7.082, *p* = 0.008) and repair (Wald = 8.358, *p* = 0.004) are correlated directly and significantly with medium happiness.

The OR calculations of the model with the reference category high happiness (Table 3) show that the probability of low happiness is 5.6 times higher among individuals who should improve clarity, 12 times higher among those who should improve repair and 3 times higher with adequate repair. Similarly, the probability of medium happiness is 3.5 times higher among individuals who should improve clarity and repair, 2.6 times higher with adequate clarity, and 2.2 times higher with adequate repair.

In addition, a receiver operating characteristic (ROC) curve was analyzed to assess the discriminative accuracy of the emotional intelligence dimensions. This allowed the identification of the cut-out points of the emotional intelligence scores beyond which high happiness becomes more likely.

**Figure 1:**
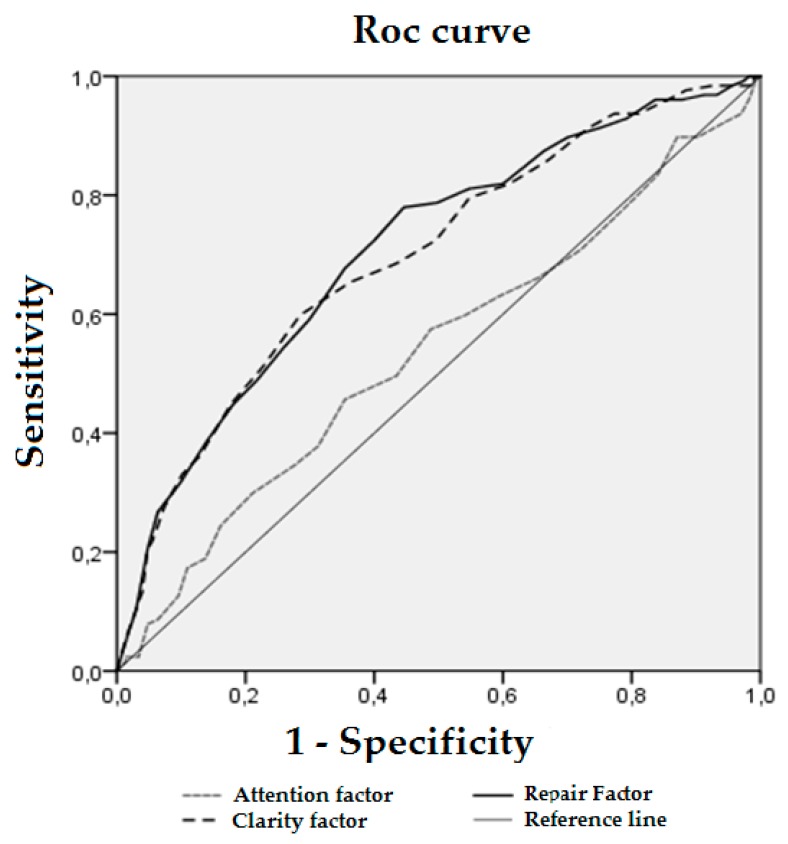
ROC curve for the TMMS-24 dimensions predicting the presence of high happiness.

In the ROC analysis, in the non-parametric case, the curve of the clarity dimension has an area below it of 0.696, 95% CI (0.644, 0.748), *p* < 0.001, and the repair dimension has below it an area of 0.707, 95% CI (0.656, 0.758), *p* < 0.001, while in the case of the attention dimension, the area below the curve of 0.536, 95% CI (0.478, 0.595), *p* = 0.206, does not provide significant information (Figure 1).

The cut-off points that simultaneously optimize sensitivity and specificity, and the separate cut-off points that optimize sensitivity and specificity of the clarity and repair dimensions are shown in Table 4.

To identify high happiness, a score of 28.5 or over in the clarity dimension simultaneously maximizes sensitivity (60%) and specificity (71%) (Youden Index = 0.314). A score of 25.5 maximizes sensitivity (72%) while specificity remains higher than expected by random, and a cut-off point of 29.5 maximizes specificity (77%) while sensitivity remains higher than expected by random (Table 4). Similarly, a point of 27.5 or over in the repair dimension simultaneously maximizes sensitivity (78%) and specificity (55%) (Youden Index = 0.333). A score of 26.5 maximizes sensitivity (79%) while specificity remains higher than expected by random, and a cut-off point of 32 maximizes specificity (76%) while sensitivity remains higher than expected by random (Table 4).

## 4. Discussion

The present study has aimed to analyze the relationship between the dimensions of emotional intelligence (attention, clarity, and repair) and happiness in a sample of adolescents and identify the cut-off points in the emotional intelligence scores, above which high happiness is more likely.

The detailed analysis of the results demonstrates a clear association between emotional intelligence and happiness. In general, these results agree with other research analyzing the association between emotional intelligence and happiness [46,56] or variables connected with it, such as personal and social adjustment [34,35,36,37,38,39,40]. To be precise, our results show that as emotional clarity and repair increase the individuals perceive themselves to be happier, and when they decrease they are less happy. No association has been found with the attention dimension. They agree with studies on adolescent populations that have found correlations between emotional clarity and repair, but not emotional attention, and variables closely related to happiness, such as well-being and psychological health [57,58,59] and quality of life [60]. 

This positive relation between happiness and emotional clarity and repair factors show that both abilities are indicators of a better emotional adjustment in adolescents [61,62,63]. Thus, the scores for clarity and repair above which happiness is maximized are situated within the established ranges for adequate emotional clarity and repair [55]. The results underscore that emotional repair has a greater association with happiness. In this line, several researchers have noted that the repair of emotions is fundamental for appropriate psychological functioning and mental health [64,65,66,67]. Adolescents with higher levels of emotional repair tend to carry out pleasant distracting activities, which can contribute to a greater feeling of happiness [68].

However, the question is: why is emotional attention not related to happiness? Although emotional attention is necessary for adaptation, paying too much attention to emotions is usually associated with maladaptive factors incompatible with happiness, such as anxiety, depression, hypervigilance, rumination, and catastrophization [32,33,51]. Therefore, from this point of view, excessive attention must be associated with low happiness. In contrast, emotional attention implies being aware of the feelings that produce pleasure (happiness) or discomfort (unhappiness). All emotions have a positive function and situations that cause discomfort are inevitable. Therefore, happiness cannot depend on their absence, but on a balance between the quantity and intensity of pleasant/unpleasant. In such a way, people who pay too much attention to their emotions and moods and do not have an adequate emotional clarity and repair would not be capable enough to understand and regulate the different emotional states [69,70,71,72].

### Study Limitations

This was a transversal study; therefore, causal associations cannot be made. Likewise, the sample used and its size restricts generalizability of results. In addition, on the one hand, using the perceptions that the individuals have of their own capacities and feelings hinders the possibility of controlling possible respondent bias. It would therefore be useful to combine their own replies to the questionnaire with tests that are able to evaluate real aptitudes to solve emotional problems. On the other, although the criterion of assigning percentiles to the groups of high, medium, and low happiness allows comparisons to be made between happier or less happy individuals, it does not guarantee the identification of the happy and unhappy individuals, and consequently the results should be interpreted with a degree of caution. Despite these limitations, this study makes interesting contributions to understanding the association between emotional intelligence and happiness.

## 5. Conclusions

The conclusions of the present study support the idea that some capacities may help to increase the attainment of health and emotional well-being during adolescence. More precisely, it has shown that as adolescents’ capacities of comprehension and emotional regulation increase, so does their subjective happiness. The important role of emotional regulation should be stressed because it is an additional factor associated with happiness.

Finally, we are aware that the educational context is the best setting in which to establish policies promoting emotional health and well-being that can reach all the students and put an end to possible inequalities in the learning of those resources. This study has attempted to determine the specific dimensions that should be focused on when teaching emotional capacities as a variable promoting happiness and emotional well-being and health during this key period of life. To be exact, the capacities of understanding and regulating emotions can be developed and increased in adolescents as a way for their perception of their own happiness to increase.

## Figures and Tables

**Table 1:** Categorization and frequencies of the study variables and descriptive statistics of the OHQ-SF questionnaire.

Variables	Categories	Frequencies		Descriptives of the OHQ-SF			
		*N*	%	M	SD	Min.	Max.
OHQ-SF	Low (P ≤ 20)	125	19.9%	3.51	0.33	2.14	3.83
	Medium (20 < P < 80)	377	59.9%	4.32	0.26	3.86	4.76
	High (P ≥ 80)	127	20.2%	5.07	0.24	4.79	5.83
TMMS-24 Attention	Little	258	41.0%	4.29	0.55	2.14	5.59
	Adequate	319	50.7%	4.32	0.57	2.45	5.83
	Excessive	52	8.3%	4.43	0.62	3.07	5.59
TMMS-24 Clarity	Should improve	251	39.9%	4.13	0.56	2.14	5.55
	Adequate	327	52.0%	4.39	0.51	2.21	5.76
	Excellent	51	8.1%	4.73	0.58	3.62	5.83
TMMS-24 Repair	Should improve	185	29.4%	4.02	0.60	2.14	5.59
	Adequate	337	53.6%	4.37	0.47	3.10	5.76
	Excellent	107	17.0%	4.64	0.53	3.34	5.83
Total		629	629	100	4.31	0.56	2.14

*M* = mean, *SD* = standard deviation. P = Percentile.

**Table 2:** Multinomial logistic regression model examining the probability of perceiving low happiness according to the degree of emotional attention, clarity, and repair.

Factors	Medium Happiness				High Happiness			
	B	OR	IC 95%		B	OR	IC 95%	
Excessive attention ^2^	0.429	0.456	0.197	1.058	−0.474	0.623	0.234	1.654
Adequate attention ^2^	0.230	0.765	0.487	1.200	−0.320	0.726	0.408	1.293
Excellent clarity ^3^	0.560	1.607	0.537	4.811	1.730 *	5.643	1.776	17.928
Adequate clarity ^3^	0.238 *	2.008	1.260	3.199	1.009 *	2.743	1.485	5.069
Excellent repair ^4^	0.422 *	3.424	1.497	7.833	2.496 *	12.133	4.590	32.074
Adequate repair ^4^	0.238 *	2.499	1.566	3.988	1.414 *	4.112	2.057	8.221

Reference categories: ^1^ Low happiness. Groups compared: ^2^ little attention: ^3^ should improve clarity; ^4^ should improve repair. * *p* < 0.05. OR: odds ratio. CI: confidence interval.

**Table 3:** Multinomial logistic regression model examining the probability of perceiving high happiness according to the degree of emotional attention, clarity, and repair.

Factors	Low Happiness ^1^				Medium Happiness ^1^			
	B	OR	IC 95%		B	OR	IC 95%	
Adequate attention ^2^	−0.474	0.623	0.234	1.654	0.311	1.364	0.626	2.972
Little attention ^2^	−0.154	0.858	0.337	2.180	0.362	1.437	0.681	3.031
Clarity should be improved ^3^	1.730 *	5.643	1.776	17.928	1.256 *	3.513	1.598	7.723
Adequate clarity ^3^	0.721	2.057	0.685	6.179	0.944 *	2.571	1.283	5.154
Repair should be improved ^4^	2.496 *	12.133	4.590	32.074	1.265 *	3.543	1.725	7.278
Adequate repair ^4^	1.082 *	2.951	1.266	6.878	0.767 *	2.154	1.280	3.622

Reference categories: ^1^ High happiness. Groups compared: ^2^ Excessive attention; ^3^ Excellent clarity; ^4^ Excellent repair. * *p* < 0.05. OR odds ratio. CI confidence interval.

**Table 4:** Sensitivity, specificity and Youden Index for the scores of the clarity and repair dimensions in the TMMS-24.

TMMS-24	Cut-off Point	Sensitivity	Specificity	Youden Index
Dimension Clarity	25.5 *	0.724	0.506	0.229
	26.0	0.705	0.535	0.240
	26.5	0.685	0.566	0.251
	27.0	0.670	0.602	0.271
	27.5	0.654	0.637	0.291
	28.0	0.626	0.676	0.303
	28.5 ***	0.598	0.715	0.314
	29.0	0.555	0.744	0.300
	29.5 **	0.512	0.773	0.285
Dimension Repair	26.5 *	0.787	0.502	0.289
	27.0	0.784	0.528	0.311
	27.5 ***	0.780	0.554	0.333
	28.0	0.752	0.577	0.329
	28.5	0.724	0.600	0.324
	29.0	0.701	0.623	0.324
	29.5	0.677	0.645	0.323
	30.0	0.634	0.673	0.308
	30.5	0.591	0.701	0.292
	31.0	0.567	0.721	0.288
	31.5	0.543	0.741	0.284
	32.0 **	0.516	0.762	0.278

*** Score that maximizes sensitivity and specificity at the same time. * Score that maximizes sensitivity. ** Score that maximizes specificity.
